# Cumulative trauma predicts hair cortisol concentrations and symptoms of depression and anxiety in pregnant women—an investigation of community samples from Greece, Spain and Perú

**DOI:** 10.1038/s41598-023-28151-9

**Published:** 2023-01-25

**Authors:** Johanna Dobernecker, Andria Spyridou, Thomas Elbert, Maggie Schauer, Susan Garthus-Niegel, Martina Ruf-Leuschner, Inga Schalinski

**Affiliations:** 1 Psychologische Hochschule Berlin, Berlin, Germany; 2 Department of Psychology, University of Konstanz, Konstanz, Germany; 3 Vivo International E.V., Box 5108, 78430 Konstanz, Germany; 4 Institute for Systems Medicine and Faculty of Medicine, Medical School Hamburg, Hamburg, Germany; 5 Department of Child Health and Development, Norwegian Institute of Public Health, Oslo, Norway; 6 Institute and Policlinic of Occupational and Social Medicine, Faculty of Medicine, Technische Universität Dresden, Dresden, Germany; 7 Department of Human Sciences, Institute of Psychology, Universität der Bundeswehr, Munich, Germany

**Keywords:** Risk factors, Psychology

## Abstract

Exposure to traumatic experiences across lifespan shapes the functioning of the hypothalamic pituitary adrenal (HPA) axis and sets individuals at risk to develop symptoms of depression and anxiety. Particularly, HPA axis regulation and the psychological health of the expectant mother have been of interest, as the health of the unborn child may be affected through changes in gestational biology. The present study investigated the potential associations between lifetime trauma, current symptoms (depression and anxiety) and hair cortisol concentrations (HCC) in pregnant women. A total of 149 pregnant women were interviewed in public outpatient clinics with varying gestational age in Greece, Spain and Perú. Lifetime trauma exposure and current symptoms of depression and anxiety were assessed. HCC was measured in scalp-near hair segments (2 cm length) reflecting cumulative cortisol secretion of the past two months. Results showed that trauma load is negatively associated with HCC and higher symptoms of depression and anxiety. There was a negative association between HCC and symptoms. The present findings support the notion that cumulative trauma exposure exerts long-lasting effects on the expectant mother’s HPA axis activity functioning and mental health and may thereby potentially create risk trajectories for the unborn child via changes in gestational biology.

## Introduction

Exposure to traumatic events across the lifespan can shape the body’s stress physiology and puts individuals at risk to develop depression and anxiety^1–4^. Trauma exposure may induce long-lasting endocrine changes through dysregulation of the hypothalamic pituitary adrenal (HPA) axis^4,5^. During pregnancy, cortisol, the hormonal end product of the HPA axis, is transmitted from mother to foetus. Given this transmission, trauma exposure^6^ as well as maternal psychopathology^7^ may negatively affect the unborn child and are thus important risk factors during pregnancy.

Recent methodological advances allow the assessment of long-term HPA axis activity by measuring cortisol concentrations in hair strands. Hair cortisol concentrations (HCC) provide a reliable retrospective measurement of HPA axis activity over several months^8^. Cortisol regulation changes as pregnancy progresses, leading to increased HCC during the third trimester^9^.

The long-term impact of traumatic experiences on HCC has been repeatedly demonstrated^10^. However, the direction of the association remains unclear, as several studies have found increased HCC^11,12^, while others report lower HCC after trauma exposure^13–16^. These heterogeneous findings may be explained by the “endocrine building block effect” put forward by Steudte-Schmiedgen et al.^4^. Converging evidence suggests a dose- and time-dependent two-staged pattern of HPA axis dysregulation after trauma exposure^5,10^. First, the body’s stress response system becomes more reactive after trauma exposure through sensitisation of the HPA axis in the initial phase, leading to a post-traumatic increase of individual baseline cortisol levels. However, this hyper-activation of the HPA axis may attenuate gradually with increasing chronicity and repeated stress exposure to ultimately result in HPA hypo-activity^5^, which in turn may predispose individuals for trauma-related symptoms^4^.

The association between traumatic events and HCC has been replicated in pregnant women^17,18^ and results suggest positive associations between exposure to traumatic events during childhood and HCC during pregnancy. Given these results and the vulnerability of traumatised children to subsequently be exposed to additional traumatisation^19–21^, considering potential lifetime exposure effects is relevant.

Exposure to traumatic events is an established risk factor for a variety of negative mental health outcomes^22–24^, including symptoms of depression and anxiety^2,25–27^. Among pregnant women childhood adversities are associated with depression and anxiety^27^ and multiple lifetime trauma exposure leads to increased maternal symptom scores^28^. However, results regarding the association between HCC and symptoms of depression or anxiety are inconsistent^28,29^ and in pregnant women no significant association has been found so far^30,31^.

The HPA axis, functioning as a primary stress response system, may link trauma exposure to adverse health outcomes^32–34^. Altered HPA axis activity in response to trauma exposure may contribute to the development of mood and anxiety symptoms^1,4^. Previously, differential associations between trauma exposure, HPA axis dysregulation, and symptoms of depression and anxiety have been reported for pregnant women^35–37^ and call for further investigation of this relationship.

As a part of Konstanzer Index (KINDEX) project, three ethnically diverse samples from Greece, Spain, and Peru were recruited with varying levels and type of trauma exposure, symptoms of depression and anxiety, as well as different socio-economic backgrounds^19^. The primary objectives of the present study were to investigate associations between (1) trauma load and HCC and (2) trauma load and symptoms of depression and anxiety and (3) to explore the relationship between HCC and symptoms of depression and anxiety in a diverse sample of pregnant women.

## Methods

### Procedure and setting

Medical staff (midwives and gynecologists) from public outpatient clinics in Greece, Spain, and Peru selected pregnant women during their regular antenatal visits and a gestational age of at least 10 weeks (e.g., Monday the first woman attending her regular antenatal visits was asked to participate in the study, Tuesday the second, Wednesday the third, …). Selected women received verbal and written information about the study by the medical staff and provided informed consent as well as agreed to be recontacted for potential validation interviews. In Spain the study was carried out in the Maternity University Hospital Virgen de las Nieves, in Granada between October 2010 and February 2011. The Peruvian study was carried out in Lima’s Suburban General Hospital Maria Auxiliadora, between March 2011 and August 2011. In Greece the study was carried out in Crete Island in the General Hospital of Chania between October 2011 and February 2012. In total, medical staff recruited N = 302 pregnant women during their regular antenatal doctor’s appointment to participate in the KINDEX project, through which we achieved the assessment of psychometric properties and validation of the structured interview KINDEX for psychosocial risk factors during pregnancy [for further details see: 38-40]. The KINDEX is a screening tool developed at the University of Konstanz originally in German, and translated and validated into Spanish and Greek through field studies conducted in Greece^38^, Spain^39^, and Perú^40^. A subsample from each country sample was randomly selected based on the date to participate in a clinical interview with a trained clinical psychologist (fluent in both Spanish and Greek) using standardized instruments validated in Greek and Spanish. During these interviews (N = 149), hair samples were collected.

The study protocol was approved by the Ethics Committee of the University of Konstanz in Germany, and by the Andalusian Public Foundation for Biosanitary Research in Oriental Andalusia in Spain (FIBAO). In all three study centres, the respective head of departments of the hospitals were thoroughly informed about the study and gave their written approval. All methods were performed based on the relevant guidelines. In accordance with the Declaration of Helsinki 2008, all participants were informed by written and verbal information about this study and all participants provided written informed consent. For participants younger than 18 years (*n* = 5 from the Peruvian sample), we retrieved additionally the written consent of their legal guardian.

### Participants

The subsample of the current study consists of 149 pregnant women in the age range of 14–44 years and between the 11th and 38th week of gestation. Participants were nationals or legal residents of Greece (*n* = 45), Spain (*n* = 65) and Perú (*n* = 39),. The age differed significantly in the cohorts, *F*_(2,142)_ = 11.92, *p* < 0.001 (Greek sample: *M* = 31, Spanish sample: M = 32, SD = 4.5, *SD* = 5.2; Peruvian sample: *M* = 26, *SD* = 8.6) as well as the gestational age (Greek sample: *M* = 23.7, *SD* = 6; Spanish sample: *M* = 33.6, *SD* = 2.4, Peruvian sample: *M* = 31.6, *SD* = 5.3). There were socio-cultural and economic differences between the three samples that have been reported previously^38–40^.

### Measures

The event list of the PDS assesses the exposure to 12 traumatic experiences across the life span that involve a serious injury, a life threat, or sexual assault to the individual themselves or witnessing such an event^41^. The following events were evaluated: serious car accident, natural disaster, violence by a member of family or other relatives, violence by a stranger, sexual violence by a member of the family or relatives, sexual violence by a stranger, combat or exposure to a war zone, other unpleasant sexual experiences, captivity, torture, life-threatening illness, and other experiences (e.g., witnessed traumatic experiences). Each traumatic experience was checked if it had been experienced at least one time during life. The overall trauma load was calculated as the sum of different traumatic event types, potentially ranging between 0 = no exposure at all and 12 = exposure to all types of traumatic events.

Current symptoms of depression and anxiety were measured with the Hopkins Symptom Checklist-25 (HSCL-25)^42^. This instrument has 15 items for depressive symptoms and 10 items for anxious symptoms. All items were rated for the last week on a scale ranging from 0 = not at all true, 1 = a little, 2 = quite a bit, and 3 = extremely. For both, symptoms of depression and symptoms of anxiety, we calculated the mean of the 10 and 15 items. The HSCL-25 showed sufficient internal consistency, Cronbach’s α = 0.78 in the Greek Sample, Cronbach’s α = 0.89 in the Spanish Sample, Cronbach’s α = 0.92 in the Peruvian Sample. We applied the cut-off > 0.75 for the mean scores of symptoms of depression and anxiety of the HSCL-25 to describe the percentage of clinical relevant symptoms^43^.

### Pre-analytical processing and measurements of HCC

A hair sample was cut with scissors of the scalp from a posterior vertex position. Each hair sample was wrapped in aluminum foil and stored in a dry place in a separate envelope. Samples were taken to Germany, before being sent via mail to the laboratory of the Chair of Biopsychology at the Technische Universität Dresden in Germany. In the laboratory, the scalp-near end of the hair strand was cut to a 2 cm length. With the restriction of 2 cm, we aimed to assure that this reflects a period within the pregnancy. Based on an average hair growth rate of approximately 1 cm per month, the present HCC in 2 cm scalp-near segments reflects the cumulative cortisol secretion over the period of the past two months^44^. For washing of hair and steroid extraction, the protocol of Davenport and colleagues^45^ was employed. In brief, each hair segment was put into a 10 ml glass container, then 2.5 ml isopropanol was added, and the tube gently mixed on an overhead rotator for three minutes. After decanting, the wash cycle was repeated two times. Then the hair samples were allowed to dry for at least 12 h. Next, the hair segments were weighed out and 7.5 mg were transferred into a 2 ml cryo vial. 1.5 ml of pure methanol was added and the steroid extraction was performed for 18 h. Samples were then spun in a microcentrifuge at 10,000 rpm for 2 min, and 1 ml of the clear supernatant was transferred into a new 2 ml glass vial. The alcohol was evaporated at 50 degrees Celsius under a constant stream of nitrogen until the samples were completely dried. Finally, 0.4 ml of water was added and the tube vortexed for 15 s. Fifty microliters were removed from the vial and used for cortisol determination with a commercially available immunoassay with chemiluminescence detection (CLIA, IBL-Hamburg, Germany). The intraassay and interassay coefficient of variance of this assay is below 8%.

### Statistical analysis and data exclusion

**Figure 1:**
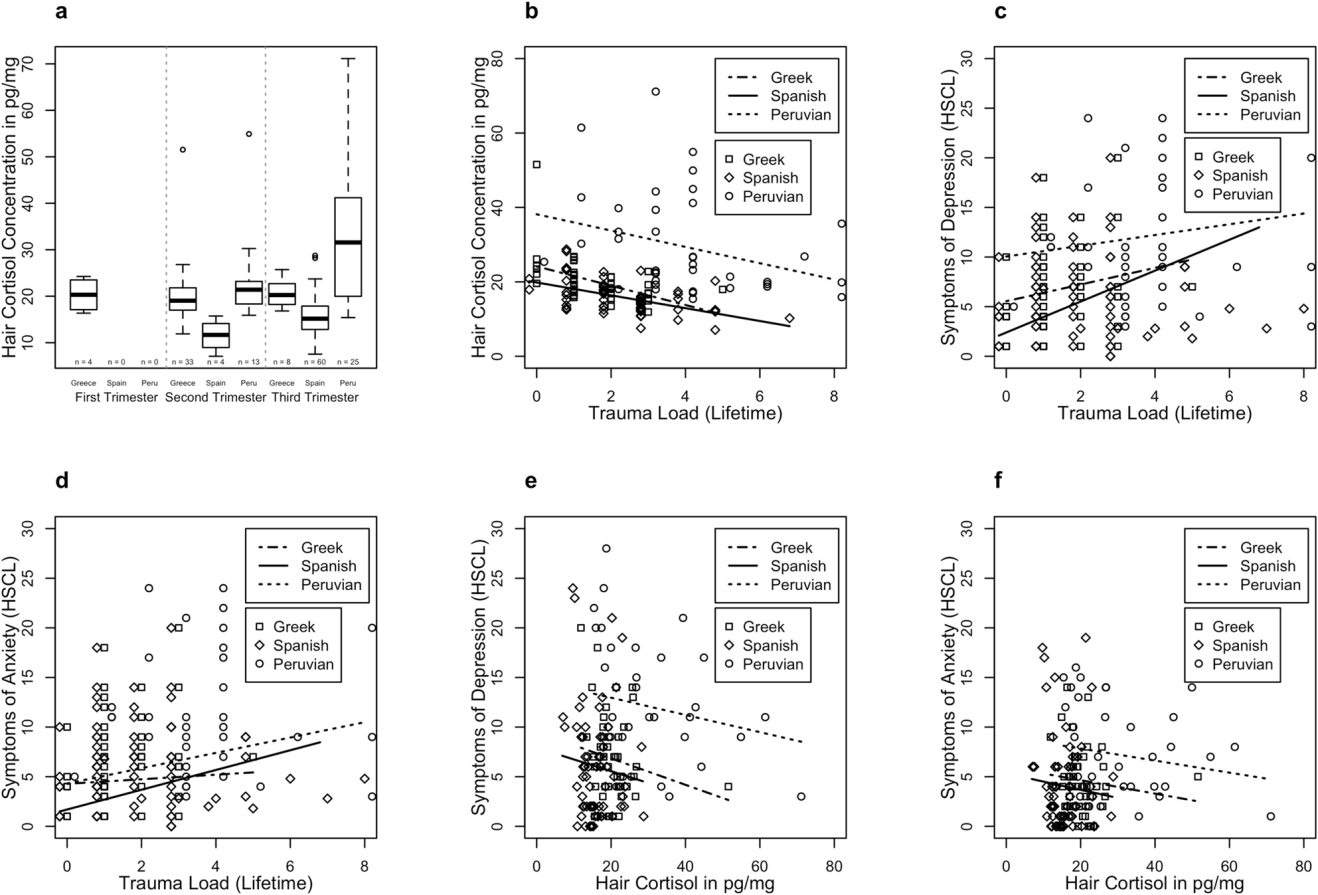
(**a**) Hair cortisol concentration (HCC) as a function of main trimester and group. (**b**) The relationship between lifetime trauma load and HCC, (**c**) trauma load and symptoms of depression and (**d**) anxiety as indicated by the Hopkins Symptom Checklist-25 (HSCL), and the relationship between HCC and (**e**) depression and (**f**) anxiety as indicated by the HSCL.

Prior to hypothesis testing, we checked for outlying HCC: we excluded all values that were above 3 *SD*s of the mean of the respective sample because external contamination could not be ruled out (see [^8^]). Data on HCC were not normally distributed, thus natural log transformation was applied to reduce skewness. Analyses were performed using R version 4.0.2. We had to remove data from *n* = 7 women: *n* = 2 women from Spain, *n* = 1 woman from Perú, and *n* = 4 women from Greece. Data from outliers will not be reported in this paper. For *n* = 2 women, there was no data available of the HSCL, and therefore data was partially excluded from the analysis. In a preparatory analysis, we checked for group differences between the Greek, Spanish, and Peruvian sample using one-way ANOVA or the Kruskal–Wallis test for continuous variables and Chi-Square for categorical variables. The HCC as a function of main trimester and group is shown in Fig. 1a. The trimester was shifted reflecting the main time frame of the past two months. For this purpose, the gestational age up to 16 weeks defined the first trimester, between 17 and 29 weeks the second trimester and from week 30 or higher the third trimester.

For the hypothesis testing, we used linear models to retrieve associations between predictor (trauma load) and outcomes (HCC, or symptoms of depression and anxiety) while controlling for the gestational week^9^ and group differences (group: Peruvian Sample, group: Greek Sample). Furthermore, we applied variance decomposition to describe the explained variance of the predictors using the R package “relaimpo”^46^.

## Results

### Preparatory analysis and descriptive statistics

All three samples differed in respect to age and gestational age (see Table 1). Cumulative cortisol secretion differed significantly between all groups (in original unit (pg/mg): *Mdn*_Greece_ = 19.2, *Mdn*_Spain_ = 14.9, and *Mdn*_Peru_ = 24.7), as well as the reported level of exposure to trauma load (Table 1). In the Greek sample the most common traumatic experiences were the exposure to serious accidents, fire or explosion (40%), other traumatic experiences (24.4%) including witnessed traumatic experiences and natural disasters (22.2%). In the Spanish sample the highest frequency was observed for the exposure to serious accidents, fire or explosion (70.3%), followed by the exposure to life-threatening illness (63.1%) and other traumatic experiences (25%), as well as the exposure to a natural disaster (25%). In the Peruvian sample, the most often reported traumatic experiences were the exposure to violence by a member of the family (55.3%), followed by serious accidents, fire or explosion (52.6%), and the exposure to violence by strangers (50%). The three samples differed in the average of reported symptoms of depression and anxiety (see Table 1). In total, *n* = 31 (20.8%) and *n* = 33 (22.4%) of the women reported symptom level above the cut-off indicating potential clinical relevant symptoms for depression and anxiety, while the Peruvian sample had higher percentages of clinical relevant symptoms compared to the other samples (see Table 1 for percentages per group).

**Table 1:** Comparison of demographic data, number of different traumatic experiences (trauma load), symptoms of depression and anxiety and hair cortisol concentration (HCC in scalp-near segment of 2 cm) of the three different samples from (1) Greece, (2) Spain and (3) Peru.

	Greece (*n* = 45)	Spain (*n* = 65)	Peru (*n* = 39)	One-way analysis of variance/Kruskal–Wallis Test/ Chi-Square Test	Group comparison	Post-hoc p-value
Demographics						
Age in years (M, SD, range)	31.0 (5.2), range (20, 42)	32 (4.5), range 23, 42)	26.1 (8.6), range (14, 42)	*F*_*2,142*_ = 11.92*, p* < 0.001	1 vs 2	> 0.999
					1 vs 3	< 0.001
					2 vs 3	0.001
Gestation age in weeks (M, SD, range)	18.0 (5.8), range (11–37)	30.8 (2.3), range (26, 37)	28.7 (5.5), range (21, 39)	*F *_*2,145*_ = 112.44, *p* < 0.001	1 vs 2	< 0.001
					1 vs 3	< 0.001
					2 vs 3	0.074
Cumulative traumatic experiences						
Trauma load (M, SD, range)	1.5 (1.1), range (0, 5)	2.6 (1.3)^a^, range (0, 7)	3.8 (1.9)^a^, range (0, 8)	*F*_*2,144*_ = 26.23,* p* < 0.001	1 vs 2	0.001
					1 vs 3	< 0.001
					2 vs 3	< 0.001
Symptoms of depression and anxiety						
HSCL: symptoms of depression (M, SD, range)	0.45 (0.30), range (0.07, 1.33)	0.41 (0.37)^a^, range (0, 1.60)	0.81 (0.44)^a^, range (0, 1.87)	*F*_*2,144*_ = 14.93*, p* < 0.001	1 vs 2	0.477
					1 vs 3	< 0.001
					2 vs 3	< 0.001
HSCL: symptoms of depression above the cut-off (> 0.75) in n (%)	7 (15.6%)	9 (14.1%)^a^	15 (39.5%)^a^	*χ*^*2*^_*(2)*_ = 10.45*, p* = 0.005	1 vs 2	0.828
					1 vs 3	0.014
					2 vs 3	0.003
HSCL: symptoms of anxiety (M, SD, range)	0.46 (0.33), range (0, 1.40)	0.40 (0.47)^a^, range (0, 1.90)	0.72 (0.45)^a^, range (0, 1.60)	*F*_*2,144*_ = 6.68*, p* = 0.002	1 vs 2	0.484
					1 vs 3	0.006
					2 vs 3	0.002
HSCL: symptoms of anxiety above the cut-off (> 0.75) in n (%)	7 (15.6%)	10 (15.4%)^a^	16 (42.1%)^a^	*χ*^*2*^_*(2)*_ = 11.37*, p* = 0.003	1 vs 2	0.992
					1 vs 3	0.007
					2 vs 3	0.003
Hair cortisol concentration						
HCC (in scalp-near segment of 2 cm) in original units in pg/mg (M, SD, range)	20.30 (5.96), range (11.90, 51.53)	15.67 (4.45), range (7.08, 15.67)	29.64 (29.63), range (15.41, 71.13)	*χ*^*2*^_*(2)*_ = 58.08*, p* < 0.001	1 vs 2	< 0.001
					1 vs 3	< 0.001
					2 vs 3	< 0.001

^a^Due to missing values data from n = 64 women Spain and n = 38 from Peru were available.

*HSCL* Hopkins Symptom Checklist-25, *HCC* hair cortisol concentration.

### Association between trauma load and HCC in pregnant women

Trauma load across the lifetime correlated negatively with HCC (*ß* = − 0.42, *p* < 0.001), while a higher gestational week was associated with higher HCC (*ß* = 0.17, *p* = 0.020). In addition, being a member of the Peruvian and Greek sample was associated with higher HCC. The overall model obtained an adjusted *R*^*2*^ of 0.51 (see Fig. 1b and Table 2, for regression coefficients and relative contribution).

**Table 2:** Summary of the regression analysis in the prediction of hair cortisol concentration (A), and HSCL symptoms of depression (B, D) and anxiety (C, E).

	Unstandardized coefficients B (SE)	Standardized coefficient β	t	p	Explained variance
A. Outcome: hair cortisol concentration (R^2^adj = 0.51, F(4, 142) = 38.6, p < 0.0001)					
Intercept	2.57 (0.17)		14.85	< 0.0001	
Dummy (1 = member of the Peruvian sample, 0 = else)	0.73 (0.06)	0.83	12.21	< 0.0001	40.8%
Dummy (1 = member of the Greek sample, 0 = else)	0.27 (0.07)	0.33	3.73	0.0003	2.8%
Pregnancy week	0.01 (0.01)	0.17	2.36	0.0199	0.7%
Trauma load (lifetime)	− 0.10 (0.02)	− 0.42	− 6.10	< 0.0001	7.7%
B. Outcome: HSCL: symptoms of depression (R^2^adj = 0.21, F(4, 142) = 10.87, p < 0.0001)					
Intercept	− 0.08 (0.23)		− 0.33	0.740	
Dummy (1 = member of the Peruvian sample, 0 = else)	0.34 (0.08)	0.38	4.31	< 0.0001	12.9%
Dummy (1 = member of the Greek Sample, 0 = else)	0.21 (0.10)	0.31	2.14	0.034	1.3%
Pregnancy week	0.01 (0.01)	0.22	1.44	0.153	0.8%
Trauma load (lifetime)	0.06 (0.02)	0.25	2.98	0.003	8.5%
C. Outcome: HSCL: symptoms of anxiety (R^2^adj = 0.12, F(4, 142) = 5.96, p < 0.001)					
Intercept	− 0.02 (0.27)		− 0.06	0.954	
Dummy (1 = member of the Peruvian sample, 0 = else)	0.24 (0.09)	0.25	2.57	0.011	5.8%
Dummy (1 = member of the Greek sample, 0 = else)	0.21 (0.11)	0.32	1.79	0.076	0.9%
Pregnancy week	0.01 (0.01)	0.22	0.87	0.387	0.3%
Trauma load (lifetime)	0.08 (0.03)	0.27	3.06	0.003	7.7%
D. Outcome: HSCL: symptoms of depression (R^2^adj = 0.20, F(4, 142) = 10.02, p < 0.0001)					
Intercept	0.63 (0.33)		1.89	0.061	
Dummy (1 = member of the Peruvian sample, 0 = else)	0.58 (0.10)	0.63	5.90	< 0.0001	17.3%
Dummy (1 = member of the Greek sample, 0 = else)	0.25 (0.10)	0.36	− 2.47	0.015	1.5%
Pregnancy week	0.01 (0.01)	0.28	1.98	0.050	1.1%
Hair cortisol concentration	− 0.25 (0.10)	− 0.23	− 2.43	0.014	2%
E. Outcome: HSCL: symptoms of anxiety (R^2^adj = 0.09, F(4, 142) = 4.81, p = 0.001)					
Intercept	0.71 (0.39)		1.82	0.072	
Dummy (1 = member of the Peruvian sample, 0 = else)	0.49 (0.12)	0.49	4.23	< 0.0001	9.2%
Dummy (1 = member of the Greek sample, 0 = else)	0.23 (0.12)	0.36	1.88	0.062	0.8%
Pregnancy week	0.01 (0.01)	0.28	1.36	0.175	0.5%
Hair cortisol concentration	− 0.25 (0.12)	− 0.22	− 2.09	0.038	1.3%

HSCL Hopkins symptom checklist-25, *HCC* hair cortisol concentration in a scalp-near segment of 2 cm length.

### Association between trauma load and symptoms of depression and anxiety

Trauma load across the lifetime was positively associated with symptoms of depression (*ß* = 0.25, *p* = 0.003), while also increased symptoms of depression were reported by women of the Peruvian and Greek sample (see Fig. 1c and Table 2). Overall, this model achieved an adjusted *R*^2^ of 0.21.

Trauma load across the lifespan was a positive predictor of symptoms of anxiety (*ß* = 0.27, *p* = 0.003). In addition to this, the Peruvian Sample obtained higher anxiety symptom levels. The overall model yielded an adjusted *R*^*2*^ of 0.12 (see Fig. 1d and Table 2).

### Association between HCC and symptoms of depression and anxiety

HCC was negatively associated with symptoms of depression (*ß* = − 0.23, *p* = 0.014), and with increasing gestational age significant more symptoms of depression were reported (*ß* = 0.28, *p* = 0.050). In addition, the Peruvian and Greek sample obtained higher symptoms of depression. In total, this model obtained an adjusted *R*^*2*^ of 0.20 (see Fig. 1e and Table 2).

Lower HCC was correlated with higher symptoms of anxiety (*ß* = − 0.22, *p* = 0.038), while the Peruvian sample also reported higher symptoms of anxiety (see Fig. 1f and Table 2). The model achieved an adjusted *R*^2^ of 0.09.

## Discussion

The goal of this study was to investigate the associations between lifetime trauma load, HCC, and symptoms of depression and anxiety in pregnant women from three different countries. Our findings support the notion that trauma exposure exerts long-lasting effects on the body’s stress response system as indicated by lower HCC with increasing trauma load in pregnant women. Furthermore, trauma load is an important vulnerability factor for lower HCC as well as higher symptoms of depression and anxiety. Consistent with previous results, a general increase in cortisol concentrations over the course of pregnancy has been observed across all subsamples^9^.

In line with the endocrine building block^4^, the present finding suggests that trauma load across the lifespan is associated with lower HCC (moderate effect size) in pregnant women from three different countries with varying degrees of trauma exposure and symptoms of depression and anxiety. Previously, negative relationships between past exposure to trauma and HCC have been reported for both individuals with and without mental illness^13^, and therefore the endocrine building block may depict a biological correlate of vulnerability. Our finding contrasts with previous findings in pregnant women, which reported positive associations between childhood trauma and HCC^17,18^. These conflicting results correspond to the current state of research, as both hyper- and hypo-activity of the HPA axis in response to lifetime trauma exposure have been repeatedly reported in non-pregnant samples^11–16^. The heterogeneity in results may be explained by stressor characteristics such as type and Timing of exposure^15^ and personal features, as well as the effect of chronic stress exposure on HPA regulation^5^.

According to Steudte-Schmiedgen et al.^4^, the chronic attenuation of HPA axis activity after trauma exposure may predispose individuals to develop trauma-related symptoms (i.e. “endocrine building block effect”). In support of this, our results show that lifetime trauma load is positively associated with symptoms of depression and anxiety (small effect size) and that these symptoms, in turn, are negatively associated with HCC (small effect size). Concerning the relationship between HCC and symptoms of depression and anxiety, results have been inconsistent^27,28^ and in pregnant women no significant association has been found so far^29,30^. Moreover, psychopathology is likely to be related to increased subjective distress, potentially contributing to HPA axis dysregulation^3–5^, however this needs further evaluation. Nevertheless, in line with the “endocrine building block effect”^4^, the present findings suggest that symptoms of depression and anxiety may arise due to chronically reduced cortisol secretion in response to trauma exposure.

The present results have shown that both, lifetime trauma load and symptoms of depression and anxiety are associated with altered maternal cortisol secretion. During the prenatal period cortisol is transmitted from mother to child via the placenta while the developing organs of the foetus display a high degree of plasticity, suggesting substantial sensitivity to variations in gestational biology^13^. Cortisol plays a crucial role for healthy fetal development, as cortisol levels usually increase adaptively over the course of pregnancy to promote appropriate fetal growth^47^. As these rising cortisol levels are essential for the organisation and programming of the fetal nervous system, changes in cortisol concentrations during gestation may result in negative consequences for developmental trajectories of the fetus^48^. Thus, alterations of the HPA axis associated with prior traumatic experiences and current symptoms of depression and anxiety may negatively affect the unborn child through direct influence on the prenatal environment. This presents one potential pathway for intergenerational transmission of maternal adversities to offspring during pregnancy, which may result in behavioural, emotional, and cognitive deficits in the offspring^49,50^. In this sense, changes in HCC during pregnancy may also lead to an increased risk of preterm delivery (e.g.,^51^) and accelerated developmental processes in the fetus (e.g., ^52^). Considering that women with a history of childhood adversities are at risk for preterm birth^53^, alterations of HPA activity during pregnancy shaped by early adversities and psychological stress burden potentially mediate this risk, however available results are mixed^51,54^. In addition to these possible direct effects of HPA axis dysregulation during gestation, postnatal maternal behaviour and psychological health related to dysregulated cortisol secretion during pregnancy may indirectly contribute to adverse consequences for offspring development^47^.

Strengths of the present study include the use of HCC as an integrative and robust measure of maternal chronic HPA axis activity, the use of three samples from different cultural backgrounds considering different levels of trauma exposure and the inclusion of lifetime cumulative trauma exposure compared to only childhood traumatic events. Furthermore, we increased the generalisability of findings by investigating the associations between trauma exposure and HPA axis activity regardless of PTSD diagnosis and by including mood and anxiety symptoms, as not all individuals develop full-scale PTSD symptoms after trauma exposure.

While this study provides further insight into the associations between lifetime trauma load, (hair) cortisol concentrations, and current symptoms of depression and anxiety in pregnant women, the results should be considered in the context of several limitations. First, the assessment of trauma exposure was based on retrospective recall which may be subject to bias. However, the PDS is a validated instrument that has been shown to be reliable^41^. Second, the present study did not differentiate specifically between different types of traumatic events ^53^ and did not consider the influence of neglect^15^. Moreover, HCC varied significantly between the three subgroups in our sample. While differences in hair growth and hair wash routine may have contributed to the variance, it seems more likely that environmental components and varying levels of trauma exposure or severity, respectively, have caused the differences^8,28^. Furthermore, the present study did not include control groups and thus the available HCC values could not be compared to those of non-pregnant women across the three samples. Additionally, methodological differences in pre-processing, hair segment lengths of 2 cm and measurement of HCC need to be considered when comparing findings across studies and laboratories. To date, a large part of research concerning cortisol levels during pregnancy has focused on examining links between childhood abuse or traumatic experiences and stress exposure during pregnancy and HCC. Swales et al.^18^ have shown that the impact of childhood traumatic events on HCC during pregnancy remains after controlling for traumatic events in adulthood and that exposure to childhood traumatic events reinforces the effect of those experienced in adulthood. Given these results and the tendency of individuals who have experienced childhood trauma to report additional traumatisation during adulthood^19–21^, the effect of exposure to traumatic events at different stages of life and their interaction on cortisol production during pregnancy deserve more attention in future research. Last, the current study had to exclude 4.5% of the HCC due to potential external contamination. It is recommended for future studies to use replicates of outlying values and test for the robustness of associations in more distal segments^11^.

Important implications can be derived from the present findings based on existing knowledge considering the long-term effects of trauma exposure on the body’s stress physiology. The relationship between past traumatic experiences and gestational endocrine regulation as well as symptoms of depression and anxiety, outlines trajectories that negatively affect women and potentially their offspring’s health^18,54,57^. Thus, our findings demonstrate the need for regular psychological screenings during prenatal care to detect the impact of past trauma on the mental health of the expectant mother. Furthermore, this calls for the development and use of trauma informed care programs in order to prevent intergenerational transmission of maternal traumatic stress^57^. For now, interventions targeting past traumatic experiences such as Narrative Exposure Therapy (NET) for trauma-related disorders^58^ may support remediation of altered endocrine regulation and the alleviation of symptoms of depression and anxiety. A recent protocol and case series demonstrates the applicability of Narrative Exposure Therapy in pregnant women^59,60^.

In conclusion, the present findings indicate that lifetime trauma load imprints the HPA axis activity and psychological well-being of the expectant mother. Past trauma exposure demonstrates an important vulnerability factor in women in the absence and presence of current mental health disorders. This justifies screenings of past traumatic experiences in routine diagnostics in prenatal care^57^. The implementation of preventive strategies and trauma-informed care are relevant in order to counterstrike the endocrine building block, alleviate maternal symptoms of depression and anxiety, and may mitigate risk trajectories for the unborn child.

## Data Availability

The datasets generated during and/or analysed during the current study are not publicly available due to German data protection requirements but are available from the corresponding author on reasonable request.
